# Perioperative Anemia in Patients Planned for Cesarean Deliveries: A Quality Improvement Audit from a Private Tertiary Care Center of Pakistan

**DOI:** 10.12669/pjms.42.5.12525

**Published:** 2026-05

**Authors:** Faraz Shafiq, Samina Ismail, Umair Aftab Baig, Iqra Wahid, Misbah Qurban Ali, Shazia Musheer, Sadique Ali Wadho

**Affiliations:** 1 Faraz Shafiq Department of Anaesthesiology, The Aga Khan University, Karachi, Pakistan; 2 Samina Ismail, Department of Anaesthesiology, The Aga Khan University, Karachi, Pakistan; 3 Umair Aftab Baig, Department of Anaesthesiology, The Aga Khan University, Karachi, Pakistan; 4 Iqra Wahid Department of Anaesthesiology, The Aga Khan University, Karachi, Pakistan; 5 Misbah Qurban Ali, Department of Anaesthesiology, The Aga Khan University, Karachi, Pakistan; 6 Shazia Musheer Department of Gynecology and Obstetrics, The Aga Khan University, Karachi, Pakistan; 7 Sadique Ali Wadho Department of Anaesthesiology, The Aga Khan University, Karachi, Pakistan

**Keywords:** Anemia, Cesarean Deliveries, Patients, Pregnancy

## Abstract

**Objective:**

Anemia in women undergoing cesarean delivery (CD) is serious concern due to consequences related to maternal and fetal health. The objective of this audit was to determine prevalence of anemia in patients planned for CD.

**Methodology:**

The retrospective record of patients from 2021 to 2023 was reviewed for age, comorbid conditions, number of pack cell transfusions done, admitting hemoglobin (Hb), discharge Hb and length of Stay (LOS) in hospital.

**Results:**

In total, data of 4,578 patients was reviewed. Nearly half (54.3%) of them were proceeded as elective case. The association of comorbid conditions was there in 55% of parturients. Amongst them gestational diabetes (55%) and hypertensive disorders (16%) were the commonest one. Overall, 44 % of patients were anemic at admission and 13 % had anemia at discharge. Only, 3.4 % of women required any sought of blood transfusion.

**Conclusion:**

The prevalence of anemia in patients undergoing CD at our center was found to be very high. Evidence based guideline for its management in antenatal period has been developed and implemented. Patient education brochure is also formulated to improve the efficacy of oral iron therapy and its being used as a part of antenatal counselling. The re-audit cycle will be planned to see the impact of implemented guidelines in reducing the prevalence of anemia in our patients planned for CDs.

## INTRODUCTION

Anemia in pregnant women is a global issue affecting almost one third of mothers.^1^ This has linked to social, economic and various health care related factors.^2^ Though considered physiological due to hemodilution, becomes pathological when daily requirement of iron is compromised.^3^ This makes iron deficiency, being the leading cause of anemia in them, global prevalence of which is around 50%.^4^ Anemia in pregnancy has impact on both maternal and fetal well-being.^5^ It is reported to be a risk factor for preterm delivery, low birth weight and higher incidence of cesarean delivery (CD).^6^ Ultimately, linked to higher morbidity and mortality.^7^ The worldwide rise in rate of CD is around 4%,^8^ while developing countries like Pakistan has even much higher rate (32%).^9^ The association of this rise with anemia makes the procedure high-risk.^10^

This is because of higher risk of infections, delayed wound healing and prolonged recovery.^11^ Understanding the prevalence of anemia is a vital part of enhancing care of obstetric patients. This is also important for incorporating effective interventions antenatally. Amongst them oral iron therapy has a stand-alone role, efficacy of which needs to be assessed thoroughly for mothers.^12^ Developing countries like Pakistan has a very high incidence of anemia (41.7-77%).^13^ This along with poor healthcare facilities and compromised nutritional status make our patients vulnerable for mentioned consequences. The primary objective of this audit was to determine the prevalence of anemia in patients had CD. The secondary objective was to implement evidence-based guidelines for its management and formulate patient education brochure to improve the efficacy of oral iron therapy.

## METHODOLOGY

Retrospective data of patients scheduled for elective or emergency CD from January 2021 to May 2023, was retrieved from operating room health management information system. A structured form was used to collect the data online from clinical record, blood bank dispensing reports and discharge summaries. It included relevant demographics, admitting hemoglobin (Hb), discharge Hb, number transfusions done, length of stay (LOS) in hospital. For the purpose of audit anemia was defined as: Hb level less than 11g/dl at the time of admission, less than 10g/dl in the second and third trimester.^14^ The severity of anemia at admission was categorized as mild, moderate and severe, based on Hb level of 10-10.9 g/dl, 7 to 9.9 g/dl and less than 7 g/dl. While at discharge it was labeled as, mild (9-9.9 g/dl), moderate (8-8.9 g/dl) and severe (less than 8 g/dl) respectively.^15^ The data was analyzed using SPSS. Frequencies and percentages were generated for descriptive statistics. As a part of this audit cycle, collaboration was made with obstetric team. Guidelines and patient education brochure were formulated for the management of anemia in patients planned for CD. The basis for both were guidelines from “*The Centre of Perioperative Care UK*”.^16^ However, the audit primarily focused on baseline data collection, and post-implementation outcomes were not assessed with current audit cycle.

### Ethical Approal:

It was obtained from Ethical Review Committee (ERC # 2024-9582-27925; dated February 6, 2024) of Aga Khan University Hospital Karachi.

## RESULTS

**Fig.1:**
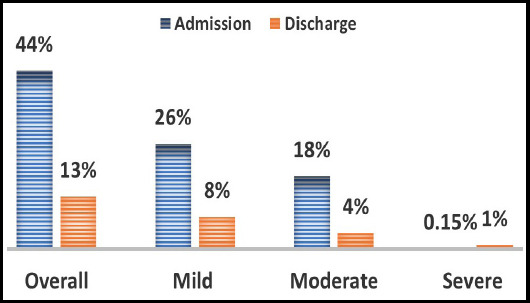
Prevalence and severity of anemia at admission and discharge.

The data of 4,578 patients was reviewed. The demographic characteristics of patients are mentioned in (Table-I). Mean age of patients was 32.79 ± 4.93. Around 54% of them were proceeded as elective case. The association of comorbid conditions was there in 55 % of mothers. Amongst them, gestational diabetes (55%) was the commonest. The mean Hb at admission was 11.07 g/dl. Overall, 44 % of percent patients were anemic at admission. The mean Hb at discharge was 11.5 g/dl and 13% of patients were anemic. The severity of anemia on scale of mild, moderate and severe, both at admission and discharge is shown in (Fig.1). Only, 3.4 % of patients required any sought of blood transfusion. The mean LOS of cohort was 4.15 days in the hospital.

**Table-I:** Demographic and related data of study cohort.

	Number(n)	Percentage (%)
1: Age Mean ±SD	32.79 ±4.93	
2: Type of Surgery		
Elective	2,462	54%
Emergency	2115	46%
3: Comorbid conditions	2500	55%
HTN disorders	410	16%
GDM	1381	55%
Others	709	28%
4: Gestational age in weeks - Median (IQR)	37(36,38)	
5: Overall need of transfusion	157	3.4%
1 unit	78	47%
2units	48	30%
More than 2 units	31	20%
5: LOS in days - Median (IQR)	4 (4,4)	

Data presented as mean ± standard deviation, median (IQR) or n (%), HTN=Hypertension; GDM=Gestational Diabetes Mellitus; CD: Cesarean Delivery; LOS= Length of Stay.

**Fig.2:**
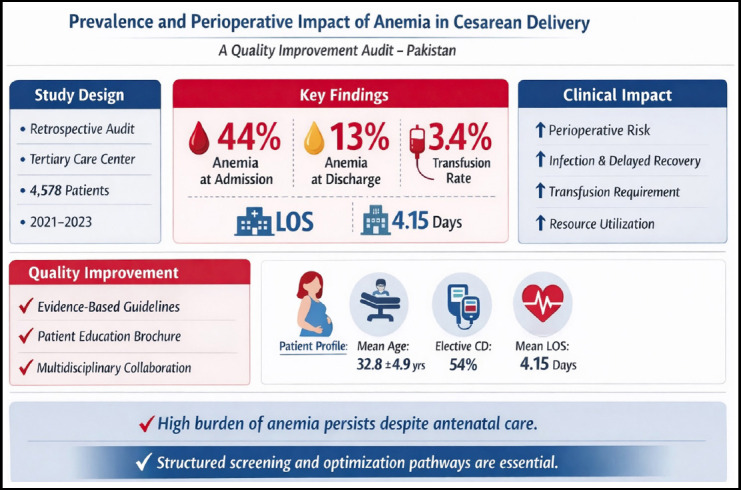
Info-graphic created using ChatGPT (OpenAI) and adapted by the authors.

## DISCUSSION

The results revealed high prevalence of anemia in our patients. The data is important for giving insight to actual scenario nationwide. The patient population in our private hospital is different as most of them belongs to urban area, well booked and maintained antenatal visits. However, scenario would be different for public sector hospitals. The results of recent cross-sectional survey from resource constraint setup showed very high prevalence of anemia (61.3%).^17^ The data from one of the biggest public sector setup showed prevalence of 71.5%.^18^ Both surveys, highlighted iron deficiency as primary cause, and reinforced the need of administrative, educational and nutritional reforms. To overcome on this alarming health issue, there is need to standardized diagnostic and prescriptive workflow. Moreover, protocol based care has been shown to improve the overall efficacy of iron therapy and admitting Hb in pregnant women.^19^ Noncompliance to iron therapy is a key concern linked to inadequate counselling, lack of understanding and associated gastrointestinal side effects.^20^ The educational reforms have been suggested the way to overcome on mentioned concerns.^21^The planning related to this audit also included formulation of guidelines and patient education brochure. Both of them have been implemented successfully and now being used in our daily clinical practice. In relation to secondary objective, although evidence-based guidelines and patient education materials were successfully developed and implemented. But current audit report did not include a follow-up phase to evaluate impact on clinical outcomes such as hemoglobin optimization or reduction in transfusion rates.

### Limitations:

This report is a retrospective audit from a single private tertiary care center, which may limit generalization to public sector hospitals and rural populations.

## CONCLUSION

The reported prevalence of anemia is very high in our tertiary care center. Evidence-based guidelines and a patient education brochure were developed and implemented. However, further studies are required to evaluate their effectiveness in improving maternal outcomes.

### Recommendations:

However, in future the re-audit cycle would focus on assessing mentioned outcomes to complete the quality improvement loop.

### Author’s Contributions:

**FS:** Substantial contributions to the conception or design of the work, analysis and interpretation of data, drafting the original manuscript and final approval of the version to be published.

**SI:** Agreement to be accountable for all aspects of work in ensuring that questions related to accuracy or integrity of any part of the work are appropriately investigated and resolved and final approval of the version to be published.

**UAB:** Acquisition, Analysis and Interpretation of data for the work and revising the manuscript critically for important intellectual content and is responsible for the accuracy of the study.

**IW SAW MQA:** Acquisition of Data. Critical Review, Analysis.

**SM:** Validation, Analysis of data and reviewing the manuscript critically for important intellectual content.
